# Effect of Sodium Nitrite, Nisin and Lactic Acid on the Prevalence and Antibiotic Resistance Patterns of *Listeria monocytogenes* Naturally Present in Poultry

**DOI:** 10.3390/foods12173273

**Published:** 2023-08-31

**Authors:** Cristina Rodríguez-Melcón, Alexandra Esteves, Javier Carballo, Carlos Alonso-Calleja, Rosa Capita

**Affiliations:** 1 Department of Food Hygiene and Technology, Veterinary Faculty, University of León, E-24071 León, Spain; 2 Institute of Food Science and Technology, University of León, E-24071 León, Spain; 3 Department of Veterinary Sciences, School of Agrarian and Veterinary Sciences, University of Trás-os-Montes e Alto Douro, 5000-801 Vila Real, Portugal; 4 Area of Food Technology, Faculty of Sciences, University of Vigo, E-32004 Ourense, Spain

**Keywords:** chicken meat, hygienic quality, *Listeria monocytogenes*, antibiotic resistance, food additives

## Abstract

The impact of treating minced chicken meat with sodium nitrite (SN, 100 ppm), nisin (Ni, 10 ppm) and lactic acid (LA, 3000 ppm) on the levels of some microbial groups indicating hygiene quality were investigated. Specifically, aerobic plate counts and culture-based counts of psychrotrophic microorganisms and enterobacteria were obtained. Additionally, the prevalence of *Listeria monocytogenes* and the resistance of 245 isolates from this bacterium to 15 antibiotics were documented. *L. monocytogenes* was isolated using the ISO 11290-1:2017 method and confirmed with polymerase chain reaction using the *lmo1030* gene. Antibiotic resistance was established using the disc diffusion technique (EUCAST and CLSI criteria). Twenty-four hours after treatment, the microbial load (log_10_ cfu/g) was reduced (*p* < 0.05) relative to controls in those samples treated with LA, with counts of 5.51 ± 1.05 (LA-treated samples) vs. 7.53 ± 1.02 (control) for APC, 5.59 ± 1.14 (LA) vs. 7.13 ± 1.07 (control) for psychrotrophic microorganisms and 2.33 ± 0.51 (LA) vs. 4.23 ± 0.88 (control) for enterobacteria. *L. monocytogenes* was detected in 70% (control samples), 60% (samples receiving SN), 65% (Ni) and 50% (LA) (*p* > 0.05) of samples. All strains showed resistance to multiple antimicrobials (between 3 and 12). In all, 225 isolates (91.8%) showed a multi-drug resistant (MDR) phenotype, and one isolate (0.4%) showed an extensively drug-resistant (XDR) phenotype. The mean number of resistances per strain was lower (*p* < 0.01) in the control samples, at 5.77 ± 1.22, than in those receiving treatment, at 6.39 ± 1.51. It is suggested that the use of food additives might increase the prevalence of resistance to antibiotics in *L. monocytogenes*, although additional studies would be necessary to verify this finding by analyzing a higher number of samples and different foodstuffs and by increasing the number of antimicrobial compounds and concentrations to be tested.

## 1. Introduction

Poultry production is one of the most widespread livestock activities worldwide, with chicken being the most commonly farmed species [1], with more than 114 million tons of chicken meat having been produced in the year 2018 [2]. This figure represents more than a third of total world meat production [3]. Some of this food is consumed in the form of meat preparations, for instance, mince. Meat preparations are defined by Regulation EC 853/2004 as fresh meat, including meat that has been reduced to fragments, which has had foodstuffs, seasonings, or additives added to it, or has undergone processes insufficient to modify the internal muscle fiber structure of the meat and thus fully eliminate the characteristics of fresh meat [4].

The extensive consumption of meat and poultry preparations justifies an interest in ensuring that these foodstuffs are safe, have a low rate of spoilage and have adequate sensorial characteristics. Products excessively contaminated with microorganisms are undesirable on both public health and financial grounds [5]. For a considerable time now, counts of viable aerobic microbiota, psychrotrophic microorganisms, and enterobacteria have been used as indicators of microbiological safety, hygiene conditions during processing, the keeping quality of poultry products, and their shelf life [5,6].

The contamination of chicken meat by pathogenic microorganisms is a worldwide concern, in view of the large quantities of this foodstuff consumed [7]. *L. monocytogenes* is a Gram-positive, rod-shaped, facultative anaerobic, non-spore-forming bacterium responsible for listeriosis, a rare but serious infection whose main route of transmission to humans is through the consumption of contaminated food [8]. Listeriosis is a zoonosis responsible for approximately 23,000 invasive infections each year worldwide [9]. During the year 2021, 2183 cases of human listeriosis were reported in the European Union, with a notification rate of 0.49 cases per 100,000 population. The age group most affected by the disease was that of those over 64 years of age, particularly the group over 84 years of age. The case fatality rate was 13.7%, the highest among all food-borne diseases in the European Union [10].

Food additives are substances that are incorporated into foodstuffs for some technological purpose, such as improving their sensorial characteristics or inactivating microorganisms, at various stages of their manufacture, transport or storage [11]. There are a range of additives that can be added to meat to reduce its microbial load and prolong its shelf life [12]. Sodium nitrite (E250) is employed in making cured meat products. Nisin (E234) is a natural polycyclic peptide compound that acts mainly against Gram-positive bacteria. Nisin is not currently approved by European legislation for use in meat and meat preparations, owing to the low acceptable daily intake (ADI), but it is allowed in certain countries, such as the USA [13]. Another widely used additive is lactic acid (E270), frequently utilized in foodstuffs for infants and young children, among other products, being applied in the amount that is enough (quantum satis). Moreover, this substance does benefit from European Union authorization for removing surface contamination from beef [14]. 

In recent decades, there has been an increase in the prevalence of bacterial resistance to antibiotics, which represents a global threat to public health since it involves a range of both different antimicrobial compounds and principal pathogenic microorganisms. The presence of antibiotic-resistant bacteria in food products is a direct threat to consumers, owing to the possibility of difficult-to-treat human infections. Furthermore, antibiotic-resistant bacteria pose an indirect danger since they are a reservoir of resistance genes that have the potential to be transferred to other microbial groups in the food chain [15].

It is estimated that by the year 2050 infections due to antibiotic-resistant bacteria will cause around ten million deaths each year worldwide [16]. This figure is in stark contrast to the 700,000 deaths attributable to antibiotic resistance in 2014 [17]. The financial consequences of antibiotic resistance are also of considerable weight, it is estimated that infections by these bacteria cost the health systems of the countries of the EU and the European Economic Area (EEA) more than EUR 1000 million each year [18].

In previous studies carried out in vitro, it was observed that contact by bacteria with low doses of biocides, including food additives, caused an increase in resistance to antibiotics in strains of various groups of bacteria [19,20,21]. However, very few studies have been carried out to determine the effect of biocides on the resistance to antibiotics of microorganisms naturally present in food [22]. It would appear that hitherto there have been no tests of the effects of sodium nitrite, nisin and lactic acid on the antibiotic resistance of *L. monocytogenes* found in chicken meat.

The main objective of this research work was to compare the effect of the three additives, sodium nitrite, nisin and lactic acid, on the microbiota of minced chicken meat from a number of retail establishments in Spain and Portugal. To this end, both before and after treatment with these additives, determinations were made of: (1) the levels of certain microorganisms indicating hygiene quality (aerobic plate counts, psychrotrophic microorganisms and enterobacteria), (2) the prevalence of *L. monocytogenes* and (3) the patterns of antibiotic resistance of this microorganism.

## 2. Materials and Methods

### 2.1. Sampling

Twenty samples of minced chicken meat weighing approximately 400 g were acquired from various outlets in León (Spain, ten samples) and Vila Real (Portugal, ten samples). All these samples were transferred in individual bags to the laboratory corresponding to each of the two locations, where they were processed immediately upon arrival.

### 2.2. Sample Processing

Eight portions of minced chicken meat were taken from each sample. The process was carried out using sterile tweezers, spoons and aluminium foil under the most aseptic conditions possible. Each of these aliquots had a weight of 25.0 ± 0.5 g.

Of the eight portions, two were treated with 2.5 mL of sodium nitrite (SN, Sigma Aldrich, Saint Louis, MO, USA) at a concentration of 1 g/L (2.5 mg in 25 g of sample; 100 ppm), another two with nisin (Ni, Sigma Aldrich) at a concentration of 100 mg/L (0.25 mg in 25 g of sample; 10 ppm), a further two with lactic acid (LA, Sigma Aldrich, St. Louis, MO, USA) at a concentration of 3% (75 mg in 25 g of sample; 3000 ppm), and the final two samples were used as controls, receiving 2.5 mL of sterile tap water. The solutions of additive were made up with sterile tap water. The concentrations used fell within the limits established by current legislation and the Codex Alimentarius. Once the additives had been incorporated, the samples were stored for 24 h at 4 °C ± 1 °C prior to analysis. Within each treatment, one sample was used to determine the levels of contamination with microorganisms indicating hygiene quality and the other to determine the presence of *L. monocytogenes*.

### 2.3. Microbiological Determinations

The minced meat aliquots used to determine the levels of microorganisms indicating hygiene quality (a total of 80, twenty for each treatment) were placed in separate sterile homogenization bags with filters, to which 225 mL of 0.1% peptone water (Oxoid Ltd., Hampshire, UK) was added. All these samples were homogenized for 120 s in a paddle homogenizer (Masticator, IUL Instruments, Barcelona, Spain). Decimal dilutions were made from the homogenate in the same diluent. Table 1 shows the culture media, incubation conditions and references used for each of the microbial groups examined.

The ISO 11290-1:2017 method was used to detect *L. monocytogenes*. The portions of minced meat, eighty in all, were homogenized in 225 mL of Half Fraser broth (Oxoid). The bags were incubated at 30 °C for 24 h. Once this time had elapsed, 100 µL aliquots were transferred to tubes with 10 mL Fraser broth. These tubes were incubated at 37 °C. After 24 h, they were streaked on the surface of Oxoid chromogenic *Listeria* agar (OCLA, Oxoid), the plates then being incubated at 37 °C for 48 h. Blue-green colonies surrounded by a halo of lighter color were deemed to be *L. monocytogenes* or *L. ivanovii*. Blue-green colonies without such a halo were considered to be *Listeria* strains belonging to non-pathogenic species. From each sample presenting blue-green colonies with a halo, five colonies were taken, these being stored at −50 °C in tryptic soy broth (TSB, Oxoid), with 20% glycerol, for later identification.

### 2.4. Identification of Listeria monocytogenes

Any colonies isolated that had presumptive *L. monocytogenes* morphology were identified using polymerase chain reaction (PCR) with primers and conditions specific to the detection of the *lmo1030* gene, as shown in Table 2.

To extract deoxyribonucleic acid (DNA), 20 µL of each isolate was inoculated into test tubes with 9 mL of TSB, these being incubated for 24 h at 37 °C. Once this period had gone by, the DNA was extracted from 1.5 mL of the culture, two centrifugation cycles were performed at 13,000 rpm for 60 s, and the sediment was left in a water bath at 100 °C for 30 min. The purity and concentration of the DNA were determined with a Nano-Drop One spectrophotometer (Thermo Fisher Scientific, Wilmington, DE, USA) using a wavelength of 260 nm. Samples whose DNA concentration was found to be in the range of 80 ng/µL to 180 ng/µL were considered suitable for carrying out the study.

PCR reactions were performed in a total volume of 25 µL, using 0.5 µM of each primer (Isogen Life Sciences, Barcelona, Spain), 0.2 mM mix of deoxynucleoside triphosphates or dNTPs (EURx, Gdansk, Poland), 1 × reaction buffer (EURx, Gdansk, Poland), 3 mM MgCl_2_ (EURx, Gdansk, Poland), 1.25 U Taq DNA polymerase (BIORON GmbH, Römerberg, Germany) and 5 μL of DNA. The final volume was made up with sterile Milli-Q water.

Amplification reactions were performed in a thermocycler from Bio-Rad (Hercules, CA, USA), programmed as follows: an initial denaturation cycle at 95 °C for five minutes, 35 amplification cycles each involving denaturation at 95 °C for 30 s, annealing at 62 °C for 30 s and elongation at 72 °C for 45 s, and finally elongation at 72 °C for 5 min.

The PCR products were separated using horizontal agarose gel electrophoresis (BIORON GmbH, Römerberg, Germany) at 1% (*w*/*v*) in 1 × Tris acetate-EDTA (TAE) buffer, stained with SimplySafe diluted to 1:10,000 (EURx, Gdansk, Poland). A transilluminator (Gel Doc EZ System, Bio-Rad) was used to visualize the electrophoresis bands. The size of each PCR product was estimated using standard molecular weight markers (10 kb DNA ladder; BIORON GmbH, Römerberg, Germany). A loading buffer consisting of glycerol (Panreac Química S.L.U., Barcelona, Spain) and bromophenol blue (Panreac Química S.L.U., Barcelona, Spain) was used. Negative controls (samples without DNA) and positive controls (DNA from *L. monocytogenes* ATCC 13932) are included in all PCR assays.

### 2.5. Antibiotic Susceptibility Testing

The susceptibility of the *L. monocytogenes* isolates to 15 antibiotics of clinical interest was determined using the disc diffusion technique on Mueller–Hinton agar (MHA, Oxoid) in accordance with the guidelines of the Clinical and Laboratory Standards Institute [27]. Discs (Oxoid) of the following 11 categories of antibiotics were used: penicillins (ampicillin -AMP, 10 µg-, oxacillin -OX, 1 µg-), cephalosporines (cefoxitin -FOX, 30 µg-, cefotaxime -CTX, 30 µg-, cefepime -FEP, 30 µg-), aminoglycosides (gentamycin -CN, 10 µg-), macrolides (erythromycin -E, 15 µg-), glycopeptides (vancomycin -VA, 30 µg-), sulphonamides (trimethoprim-sulfamethoxazole -SXT, 25 µg-), ansamycins (rifampicin -RD, 5 µg-), tetracyclines (tetracycline -TE, 30 µg-), amphenicols (chloramphenicol-C, 30 µg-), fluoroquinolones (ciprofloxacin -CIP, 5 µg-, enrofloxacin -ENR, 5 µg-), and nitrofuran derivatives (nitrofurantoin -F, 300 µg-). After incubation for 24 h at 37 °C, inhibition halos were measured, and strains were classified as susceptible, intermediate (having reduced susceptibility), or resistant according to the criteria of EUCAST [28] and CLSI [27]. Antibiotic resistance patterns were established and classified on the basis of the criteria defined by the European Centre for Disease Prevention and Control (ECDC) and the Centres for Disease Control and Prevention (CDC) of the USA for bacteria of interest for public health. These norms include references to “multidrug-resistant” (MDR), “extensively drug-resistant” (XDR), and “pan-drug-resistant” (PDR) phenotypes. The MDR phenotype is defined as an acquired absence of susceptibility to at least one antibiotic from each of three or more categories of antimicrobials. The XDR phenotype is explained as a lack of susceptibility to at least one antimicrobial agent from all but two or fewer antimicrobial categories. Finally, the PDR phenotype involves an absence of susceptibility to all agents in all antimicrobial categories [29].

### 2.6. Statistical Analysis

To carry out the statistical analysis, the microbial counts were transformed into logarithmic units (log_10_ cfu/g) and compared using analysis of variance (ANOVA) techniques, with the separation of means being achieved with Duncan’s test. The prevalence of *L. monocytogenes* and the prevalence of resistant isolates were compared using the two-tailed Fisher’s exact test. To compare the average number of antibiotics to which the isolates were resistant, the Mann–Whitney U test was performed. Significant differences were set at a probability level of 5% (*p* < 0.05). Statistical analysis was performed using Statistica^®^ 8.0 software (Statsoft Ltd., Tulsa, OK, USA).

## 3. Results

### 3.1. Microbial Counts

The microbial loads in each of the groups of samples analyzed are shown in Table 3. The levels of aerobic plate counts (APCs) ranged between 5.51 ± 1.05 log_10_ cfu/g in samples treated with LA and 7.53 ± 1.02 log_10_ cfu/g in the control samples. With regard to psychrotrophic microorganisms, the counts ranged between 5.59 ± 1.14 log_10_ cfu/g (LA) and 7.13 ± 1.07 log_10_ cfu/g (control). Finally, the levels of enterobacteria were between 2.33 ± 0.51 log_10_ cfu/g with LA treatment and 4.23 ± 0.88 log_10_ cfu/g for controls. Only treatment with LA achieved a reduction in microbial levels relative to the untreated control samples.

### 3.2. Prevalence of Listeria monocytogenes

On the OCLA medium, colonies with characteristic morphology of *L. monocytogenes* (green with halo) were detected in fourteen samples (70.0%) among controls, twelve (60.0%) of those treated with SN, thirteen (65.0%) of those receiving Ni treatment, and ten (50.0%) of the samples treated with LA. No significant differences (*p* > 0.05) were observed in the prevalence of *L. monocytogenes* in the different types of samples, although there was a tendency for the treated samples to present a lower prevalence of the microorganism than the control samples (58.3% overall as against 70.0%).

The *lmo1030* gene was detected in all colonies with the characteristic morphology of *L. monocytogenes* isolated from OCLA, five from each positive sample, amounting to a total of 245.

### 3.3. Antibiotic Susceptibility

**Figure 1:**
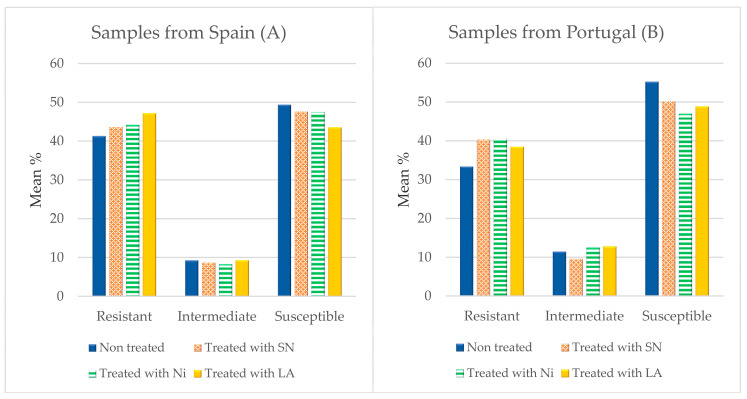
Mean percentages of resistance, reduced susceptibility (intermediate) or susceptibility in *L. monocytogenes* isolates from samples of Spanish (**A**) and Portuguese (**B**) chicken mince by treatment received. SN = sodium nitrite; Ni = nisin; LA = lactic acid. Data for control samples are from Rodríguez-Melcón et al. [30].

The susceptibility of the 245 *L. monocytogenes* isolates to a panel of 15 clinically important antibiotics was determined. The percentage of susceptible strains, those with reduced susceptibility (intermediate) or resistant, when the whole range of antibiotics is taken together, is shown in Figure 1. The presence of strains resistant to antibiotics in control samples was 41.33% in the case of the isolates from chicken obtained in Spain and 33.33% for those from Portugal (*p* > 0.05). After treatment with additives for 24 h, an increasing trend (*p* > 0.05) in the percentage of resistances was observed, with figures of 43.62% (samples treated with SN), 44.17% (Ni) and 47.47% (LA) for the samples of Spanish origin, and percentages of 40.27% (SN), 40.53% (Ni) and 38.40% (LA) for those from Portugal, as shown in Figure 1. The average number of resistances per strain was lower (*p* < 0.01) in the control samples (5.77 ± 1.22) than in those receiving treatment (6.39 ± 1.51). When samples were grouped by treatment, the figures were 6.33 ± 1.22 resistances per strain (samples treated with SN), 6.42 ± 1.43 (Ni) and 6.42 ± 1.82 (LA) (*p* > 0.05).

**Figure 2:**
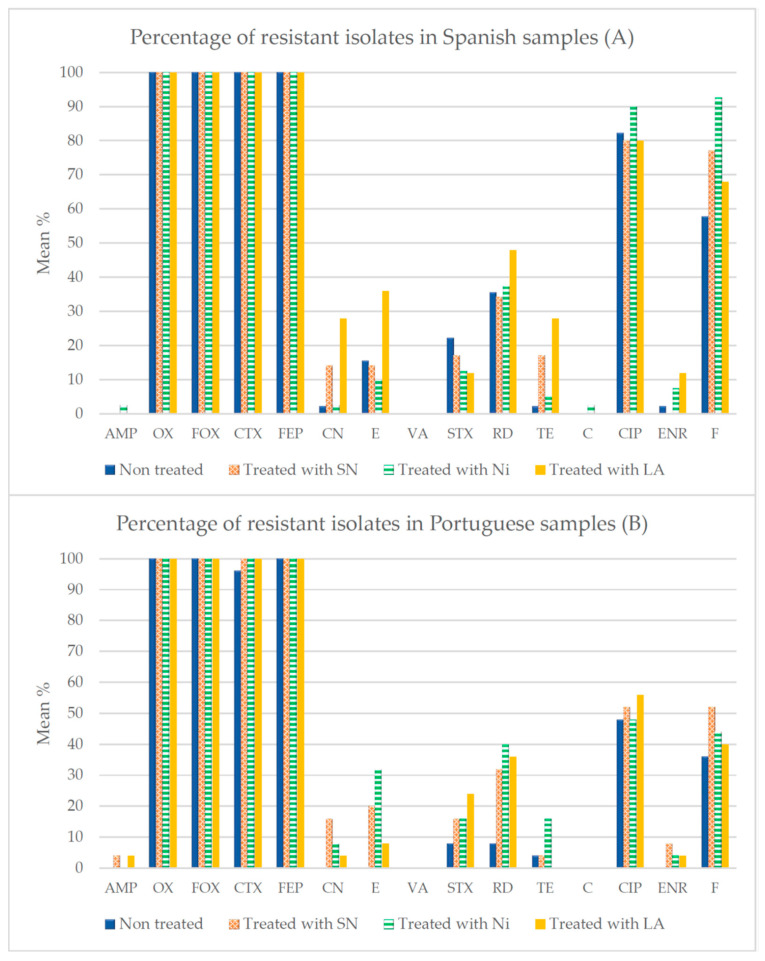
Mean percentages of resistance for each antibiotic used in *L. monocytogenes* isolates from samples of chicken mince of Spanish (**A**) and Portuguese (**B**) origin, treated with additives or untreated. Data for control samples are from Rodríguez-Melcón et al. [30]. AMP (ampicillin, 10 µg), OX (oxacillin, 1 µg), FOX (cefoxitin, 30 µg), CTX (cefotaxime, 30 µg), FEP (cefepime, 30 µg), CN (gentamycin, 10 µg), E (erythromycin, 15 µg), VA (vancomycin, 30 µg), SXT (trimethoprim-sulfamethoxazole, 25 µg), RD (rifampicin, 5 µg), TE (tetracycline, 30 µg), C (chloramphenicol, 30 µg), CIP (ciprofloxacin, 5 µg), ENR (enrofloxacin, 5 µg), and F (nitrofurantoin, 300 µg). Non-treated samples (control), SN (samples treated with sodium nitrite), Ni (samples treated with nisin), LA (samples treated with lactic acid).

Figure 2 shows the percentages of strains resistant to each of the antibiotics examined after treatment of the samples with SN, Ni and LA. The data for untreated samples are from Rodríguez-Melcón et al. [30]. Major differences were found between compounds, and it was observed that more than 95% of isolates were resistant to OX, FOX, CTX and FEP. Other antibiotics to which high rates of resistance were observed were CIP and F, in strains isolated from minced meat purchased both in Spain and in Portugal.

After the treatments with additives, several changes in the resistance of the strains were detected. Thus, after 24 h of contact with these substances, in the samples from Spain, an increase was observed in the number of strains resistant to CN (*p* < 0.01) and to TE (*p* < 0.01) when treated with SN or with LA, to E (*p* < 0.05) and to ENR (*p* < 0.01) with LA treatment, and to F (*p* < 0.01) in the case of treatments with SN and with Ni. In the strains of Portuguese origin, after application of the additives, there was increased resistance to CN (*p* < 0.01) with SN and Ni treatments, to E (*p* < 0.01) and to RD (*p* < 0.001) with all three substances, to SXT (*p* < 0.01) with LA, to TE (*p* < 0.01) with Ni, and to ENR (*p* < 0.01) and F (*p* < 0.05) when treatment was with SN.

**Figure 3:**
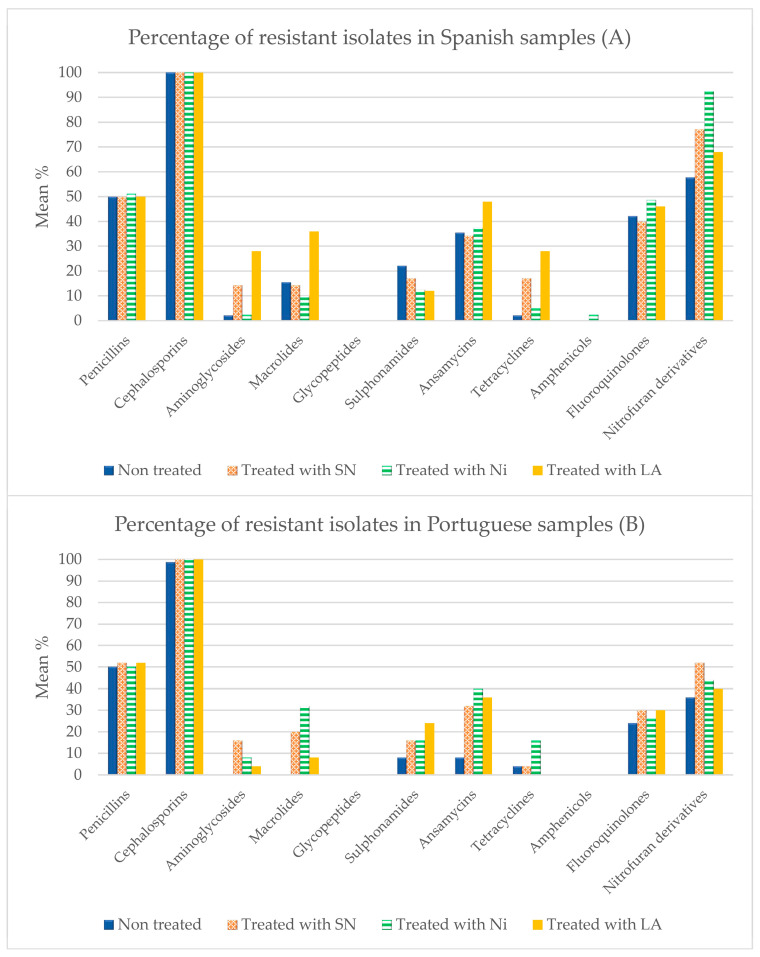
Mean percentages of resistance for each class of antibiotics used in *L. monocytogenes* isolates from samples of chicken mince of Spanish (**A**) and Portuguese (**B**) origin, treated with additives or untreated. Data for control samples are from Rodríguez-Melcón et al. [30]. Penicillins (ampicillin -AMP, 10 µg-, oxacillin -OX, 1 µg-), cephalosporines (cefoxitin -FOX, 30 µg-, cefotaxime -CTX, 30 µg-, cefepime -FEP, 30 µg-), aminoglycosides (gentamycin -CN, 10 µg-), macrolides (erythromycin -E, 15 µg-), glycopeptides (vancomycin -VA, 30 µg-), sulphonamides (trimethoprim-sulfamethoxazole -SXT, 25 µg-), ansamycins (rifampicin -RD, 5 µg-), tetracyclines (tetracycline -TE, 30 µg-), amphenicols (chloramphenicol -C, 30 µg-), fluoroquinolones (ciprofloxacin -CIP, 5 µg-, enrofloxacin -ENR, 5 µg-), and nitrofuran derivatives (nitrofurantoin -F, 300 µg-). Non-treated samples (control), SN (samples treated with sodium nitrite), Ni (samples treated with nisin), LA (samples treated with lactic acid).

Figure 3 shows the percentages of strains resistant to each class of antibiotic examined after treatment of the samples with SN, Ni and LA. In the samples from Spain, an increase was observed in the percentage of strains resistant to aminoglycosides (*p* < 0.01) and to nitrofuran derivatives (*p* < 0.01) when treated with SN, to nitrofuran derivatives (*p* < 0.001) with Ni treatment, and to aminoglycosides (*p* < 0.001) and macrolides (*p* < 0.01) in the case of treatment with LA. In the strains of Portuguese origin, after application of the additives, there was increased resistance to aminoglycosides (*p* < 0.001), macrolides (*p* < 0.001) ansamycins (*p* < 0.001) and nitrofuran derivatives (*p* < 0.05) with SN treatment, to amynoglycosides (*p* < 0.01), macrolides (*p* < 0.001), ansamycins (*p* < 0.001) and tetracyclines (*p* < 0.01) with Ni, and to macrolides (*p* < 0.01), sulphonamides (*p* < 0.01) and ansamycins (*p* < 0.001) when treatment was with SN.

The different patterns of resistance to antibiotics found in the samples can be observed in Table 4 for the control samples, which come from the study of Rodríguez-Melcón et al. [30], and Table 5, Table 6 and Table 7 for the samples treated with additives. One isolate from control samples showed resistance to three antibiotics, eight isolates to four antibiotics, and sixty-one isolates had a multidrug-resistant (MDR) phenotype (between five and eight antibiotics). In the samples treated with SN, two isolates were resistant to four antibiotics, and fifty-eight isolates showed a MDR phenotype (between five and ten antibiotics). In the samples treated with Ni, three isolates were resistant to four antibiotics, and sixty-two were classified as MDR (between five and ten antibiotics). Finally, strains isolated from LA-treated samples were resistant to four antibiotics (six isolates), showed an MDR phenotype (between five and ten antibiotics; 43 isolates) or showed an extensively drug-resistant (XDR) phenotype (resistant to twelve antibiotics in nine categories; one isolate). None of the strains tested had a pan-drug-resistant (PDR) phenotype.

The most frequently observed pattern was OX-FOX-CTX-FEP-CIP-F. This was seen in 14 isolates from the control samples, in 18 from those treated with SN, 18 with Ni and 8 with LA. Other frequently repeated patterns are OX-FOX-CTX-FEP-RD-CIP-F for NS, with a total of seven isolates (six of Spanish origin and one of Portuguese), OX-FOX-CTX-FEP-RD-CIP-F for Ni, with eight isolates in total, all from Spain, and OX-FOX-CTX-FEP and OX-FOX-CTX-FEP-CIP for LA, where the total was six isolates each. It is noteworthy that one isolate of *L. monocytogenes* resistant to 12 antibiotics was found in the samples treated with LA.

## 4. Discussion

### 4.1. Microbial Counts

The APC observed in the control samples fell within the range of values (log_10_ cfu/g) noted in poultry meat preparations acquired from retail establishments in earlier studies. Figures recorded for Spain lay between 6.29 ± 0.64 and 7.28 ± 0.51 [6], and in other countries, a value of 6.05 ± 0.18 was reported by Lerasle et al. [31].

With regard to psychrotrophic microorganisms, the meat samples presented acceptable quality according to European Regulation EC 2073/2005 [32]. Apart from such legal requirements, guidelines and recommendations have been developed to monitor the microbiological quality of meat by the International Commission on Microbiological Specifications for Foods [33] and by the British Institute for Food Science and Technology [34]. The counts of psychrotrophic microorganisms recorded in this current research work did not match these international guidelines for good manufacturing practices, where the desirable levels should be 5 log_10_ cfu/g or below, and indeed were close to, or above, the maximum acceptable level of 7 log_10_ cfu/g. It should be noted that microbial levels greater than 7 log_10_ cfu/g or cm^2^ are generally associated with incipient sensory disturbances [33].

It is likely that during the time the mince was in refrigerated storage at the retail outlet, prior to acquisition, levels of psychrotrophic microorganisms increased relative to initial values, as observed in previous work [5,35]. On this point, it should be noted that the exact length of time the meat remained in the establishment before purchase is not known.

The enterobacteria counts in the control samples, at 4.23 ± 0.88 log_10_ cfu/g, lay above the limits used as microbiological criteria for free-range poultry in Spain, which are set at 2 log_10_ cfu/g [36].

The additives tested, specifically SN, are used as preservatives in numerous types of processed meats, being employed to slow, and in some cases to reduce, the growth of pathogenic and spoilage microorganisms through mechanisms that include the interruption of oxygen uptake and oxidative phosphorylation, as well as the inhibition of enzymes such as aldose, critical in bacterial metabolism [37]. In the present study, no significant differences were observed between the counts obtained in the control samples and the samples treated with SN for any of the microbial groups studied. In contrast, other authors have observed that this compound does reduce microbial levels in meat. Thus, Wójciak et al. [38] observed that in beef and pork mince, the concentration of SN applied was inversely proportional to the levels of enterobacteria, which ranged between 2.75 log_10_ cfu/g and 6.03 log_10_ cfu/g. The results obtained by these authors revealed that the addition of 100 mg/kg of SN, the concentration used in the study being reported here, would be adequate for minced meat, as this amount did not modify the sensory characteristics of the product as compared to the control sample [32]. On the other hand, Gunvig et al. [39] observed the absence of growth of *Clostridium botulinum* in certain products, such as cooked meats, after the application of SN at concentrations between 72 ppm and 150 ppm. Other authors [40,41] have also indicated that nitrites are very effective as bacteriostatic and bactericidal agents to inhibit or regulate the development of bacteria to varying degrees in meat products. This compound has been shown to prevent the growth of *Clostridium botulinum* and slow the growth of pathogenic microorganisms such as *Listeria monocytogenes*, *Bacillus cereus*, *Clostridium perfringens*, *Staphylococcus aureus*, and *Escherichia coli* at levels found in various meat products such as cured meats and poultry products [42]. For their part, González and Díez [43] demonstrated that SN at concentrations between 50 ppm and 150 ppm caused a reduction in the *Enterobacteriaeae* count in a typical Spanish sausage such as “chorizo”. In addition, nitrites have shown their antimicrobial effectiveness on the microbiota present in meat [44]. It should be noted that variations in the results observed between studies may be partly due to differences in the procedures used to apply antimicrobial treatments.

Similarly, Ni did not achieve any significant reduction in microbial counts in the current study. In contrast, other authors have observed reductions in APC in different types of meat when nisin [45], or a combination of nisin with tea polyphenols and chitosan [46], was applied. It should be noted that nisin has been shown to have greater antimicrobial potential in meat preparations when in combination with other substances [47]. Yuste et al. [48] pointed out that the addition of concentrations of 100 or 200 ppm of nisin together with a 450 MPa pressurization in samples of mechanically separated chicken meat significantly reduced the microbial load present. It should be kept in mind that the current research used lower concentrations of nisin (10 ppm), and this may have influenced the limited effectiveness observed for this compound.

The samples treated with LA were those that presented the lowest levels (*p* < 0.05) of all the microbial groups studied. Recent research [49] demonstrated the efficacy of this compound at a concentration of 3%, the same as used in the present study, as a decontaminant for beef, with minimal modifications to sensory parameters. The antimicrobial efficacy of LA in meat has also been reported by the European Food Safety Authority [50,51]. Organic acids, such as LA, have also been widely used as antimicrobial decontaminants on chicken carcass surfaces due to availability, cost-effectiveness, ease of use, and antimicrobial potential [52]. The mode of action of LA occurs with the undissociated form, which crosses the cytoplasmic membrane, lowers the intracellular pH and causes bacterial inactivation [53].

### 4.2. Prevalence of Listeria monocytogenes

A total of 245 *L. monocytogenes* isolates were obtained from the samples of chicken mince purchased in northwestern Spain and northern Portugal, both treated and untreated with food additives. A 100% agreement was observed between the chromogenic media plating (OCLA) method and PCR since all the isolates displaying the typical *L. monocytogenes* morphology of blue-green colonies with a lighter halo were identified as this specific microbial species via PCR. These results are similar to those of other studies, in which a high level of agreement was observed between classic techniques and PCR in detecting *L. monocytogenes*, values recorded being 100% [54], 98.4% [55] or 89.4% [56]. 

A total of 70% of the untreated samples of mince (control) were contaminated with *L. monocytogenes*. These values are similar to those recently observed in chicken cuts from northwestern Spain, with 56.0% recorded by Panera-Martínez et al. [57]. Other studies detected the prevalence of *L. monocytogenes* in chicken meat from northwestern Spain of 24.5% in chicken legs [58] and 32.0% in chicken carcasses [59]. Other data for prevalence found in the bibliography vary considerably. Figures reported range from 0.0% to 58.0% [60,61], 0.2% to 2.5% [62], 4.3 % to 7.1% [63], 8.6% to 44.2% [64], 11.4% to 14.1% [65], 12.7% [66], 15.8% [67], 17.9% [68], 18.0% [69], 18.2% [70], 19.2% [71], 19.3% [72], 20.0% [73], 22.2% [74], 26.4% [75], 34.0% [76], 36.1% [77], 38.0% [78], 38.2% [79], 40.0% [80], 41.0% [81] or 45.0% [82]. It should be noted that the differences between studies may be due, at least in part, to the type of samples analyzed, which consist of cuts or carcasses in most of the works consulted. In the present study, minced meat was used, so a higher prevalence was to be expected. This is because the processing and handling necessary to manufacture meat preparations enhance the risks of contamination with different microorganisms, including *L. monocytogenes* [68]. *L. monocytogenes* is a ubiquitous bacterium that can access food processing facilities, for example, butcher shops, and persist for long periods of time [83] since its high resistance to adverse conditions makes its eradication difficult [84,85]. It has been shown that the implementation of good hygienic practices (GHPs), together with adequate cleaning and disinfection procedures, are effective measures to reduce the contamination of food with this microorganism [85].

The prevalence of *L. monocytogenes* in the treated samples, at 50% to 65%, tended to be lower (*p* > 0.05) than in the control samples (70%). Other authors [86] observed a significant reduction in the prevalence of *L. monocytogenes* present in ham samples treated with sodium nitrite, but only from the twelfth day of storage onwards.

As for treatment with nisin, several studies have shown the antimicrobial efficacy of this compound, with reductions in *L. monocytogenes* counts (log_10_ cfu/g) observed in meat after this substance was applied. Thus, Ariyapitipun et al. [87] found *L. monocytogenes* levels of 3.53 log_10_ cfu/g in untreated beef samples and 2.21 log_10_ cfu/g in samples treated with the compound in question. Solomakos et al. [88] recorded 6.10 log_10_ cfu/g in their control sample and 4.40 log_10_ cfu/g in nisin-treated beef samples, and Raeisi et al. [89] observed a reduction from 8.90 log_10_ cfu/g in untreated meat to 6.60 log_10_ cfu/g in chicken meat samples treated with nisin in combination with cinnamon. In contrast, Pawar et al. [90] did not find any reductions between untreated control samples of buffalo meat and portions treated with nisin. The antimicrobial effect of nisin against *L. monocytogenes* has also been observed in processed meats. Samelis et al. [91] found a reduction for *L. monocytogenes* of one log_10_ cfu/g in bologna sausage (“baloney”) samples. Other authors detected a reduction of 0.10 log_10_ cfu/g in turkey sausages [92], 1.52 log_10_ cfu/g in turkey ham [93], and 6.6 log_10_ cfu/g in turkey sausages [94]. Finally, lactic acid has also been shown to be effective at a 2% concentration in reducing *Listeria* levels [95].

### 4.3. Susceptibility to Antibiotics

The presence in poultry meat of bacteria with multiple resistances to antibiotics of clinical importance has been previously studied [35,58,96,97], with high levels of resistance being observed, as in the present study. This implies a crucial challenge for public health because in cases of infection by this microorganism, many antimicrobials commonly used in clinical practice would be invalidated as therapeutic options.

In the present study, all the strains of *L. monocytogenes* showed multiple resistances, a state of affairs observed in previous research [57]. The average number of resistances per strain was also similar, the figure of 5.83 ± 1.64 in the study by Panera-Martínez et al. [57] being very close to the 5.77 ± 1.22 noted in the current research. The high percentage of isolates with an MDR phenotype observed in the present report is also a finding coincident with previous research works [57]. 

The considerable prevalence of resistance to OX, FOX, CTX, FEP, CIP and F recorded here was also detected in previous research carried out with meat and poultry preparations from northwestern Spain [16,49,50]. It should be noted that some of the antibiotics to which high levels of resistance were observed are classified as “critically important” (AMP, CTX, FEP, VA, RD, CIP), “highly important” (OX, FOX, TE, C) or “important” (F) in human medicine [98]. In the list of the World Organization for Animal Health, AMP, OX, TE and CIP are considered “critically important” antibiotics, and RD “of high importance” in veterinary medicine [99]. Furthermore, some of these substances are used for the treatment of human listeriosis, with beta-lactam antibiotics, usually ampicillin, administered alone or in combination with gentamycin being the drugs of first preference. In patients allergic to beta-lactams, possible alternatives include erythromycin, vancomycin, trimethoprim-sulphamethoxazole, and fluoroquinolones. On occasion, listeriosis is treated with rifampicin, tetracycline and chloramphenicol [16,100]. The trend to increase resistance to some of these antibiotics (gentamicin, erythromycin, trimethoprim–sulfamethoxazole or enrofloxacin) observed after treatment with food additives is worrying. It is noteworthy that one strain showed an extensively drug-resistant phenotype after LA treatment.

The presence of resistance to antibiotics in meat is an expected result due to the wide use of these substances in animal production during recent decades, with penicillins and tetracyclines being the most common antimicrobials used in food-producing animals in both Spain and Portugal [101,102]. On these lines, it should be noted that the selective pressures from the use of antibiotics, particularly when employed incorrectly in sub-inhibitory doses, have been identified as the main cause of the marked increase in the prevalence of antibiotic resistance that has taken place during recent decades [15]. 

The consumption of antibiotics has been declining in the European Union in recent years. Thus, sales of antimicrobial veterinary medicinal products for food-producing animals in Spain decreased from 259.5 mg/PCU (population correction unit) in 2010 to 154.3 mg/PCU in 2020. The data for Portugal were 178.9 mg/PCU (2010) and 175.8 mg/PCU (2020) [102]. In addition, on 28 January 2022, new laws (Regulation (EU) 2019/4 on medicated feed and Regulation (EU) 2019/6 on veterinary drugs) entered into force in the European Union, prohibiting the routine feeding of farm animals with a diet of antibiotics. However, it must be taken into account that despite the fact that the consumption of antibiotics is decreasing, it is expected that resistance will continue to exist due to cross-resistance or co-resistance mechanisms [103].

Results in the present study suggest there is a trend of increasing resistance to several antibiotics after the application of food additives, which is a worrying state of affairs. As in the present study, other authors have also recorded an increase in the prevalence of antibiotic resistance when exposing strains of different bacterial species to low doses of biocides, whether disinfectants, decontaminants or additives [16,21,104,105]. It is possible that the increase in overall resistance is due to the cross-resistance or co-resistance mechanisms or to the triggering of a genetic SOS response, as previously suggested [15].

This is novel work that suggests the potential of food additives to increase the prevalence of antibiotic resistance in *L. monocytogenes*. However, this study has some limitations, so additional research is needed to corroborate these results. First of all, the number and type of samples analyzed, as well as the concentrations of additives, must be expanded. If the findings of this research work are confirmed, the effect of additives on the resistance to antibiotics should be determined previously to the authorization of a new compound.

## 5. Conclusions

Minced chicken meat is a food of questionable microbiological quality, in light of the high levels of microorganisms indicative of poor hygiene quality and the considerable presence of *L. monocytogenes*, which was detected in 70% of the samples examined. Likewise, the extensive prevalence of resistance to antibiotics in the strains of *L. monocytogenes* analyzed is worthy of note. These results point to a need to follow correct hygiene practices when handling chicken mince so as to avoid cross-contamination events or insufficient cooking, with the aim of reducing risks to consumers.

Of the three additives studied, sodium nitrite, nisin and lactic acid, the third showed the most effectiveness as an antimicrobial agent. Under the conditions tested, it alone was able to reduce levels of aerobic plate counts, psychrotrophic microorganisms and enterobacteria relative to untreated samples. Moreover, it achieved the strongest tendency to a decrease in the presence of *L. monocytogenes*, with figures of 70% in control samples as against 50% in samples treated with lactic acid. An increase in the levels of resistance to antibiotics of the strains of *L. monocytogenes* was observed after samples were treated with the additives under test. These results suggest that before any food additive is authorized, studies should be carried out to determine the potential capacity of the compound in question to contribute to any increase in resistance to antibiotics. However, the results in this present report must be taken with caution, in view of the limited number of samples analyzed, so additional studies are required in order to confirm these findings.

## Figures and Tables

**Table 1:** Culture media, temperature and time of incubation and references used for each microbial group tested.

Microbial Group	Culture Media	Incubation		References
		Time	Temperature (°C)	
Aerobic plate counts (APCs)	PCA ^1^	3 days	30 °C	[23]
Psychrotrophic microorganisms	PCA ^1^	10 days	7 °C	[24]
Enterobacteria	VRBGA ^2,3^	24 h	37 °C	[25]

^1^, plate count agar, spread plate technique (0.1 mL); ^2^, crystal violet, neutral red, bile salts, glucose, agar, ^3^ pour-plate technique (1 mL) with addition of overlay. The inoculations were performed in duplicate.

**Table 2:** Gene and primers used to identify presumptive strains of *L. monocytogenes* via PCR.

Gene	Primers	Sequency (5′ → 3′)	Annealing Temperature (°C) (Product Size, bp)	Reference
*lmo1030*	Lmo1030-F	GCTTGTATTCACTTGGATTTGTCTGG	62 (509)	[26]
	Lmo1030-R	ACCATCCGCATATCTCAGCCAACT		

**Table 3:** Microbial counts (log_10_ cfu/g) in chicken mince, untreated and treated with various food additives.

Microbial Group	Food Additive			
	Control (Without Treatment)	Sodium Nitrite (SN)	Nisin (Ni)	Lactic Acid (LA)
Aerobic plate counts	7.53 ± 1.02 a	7.01 ± 1.23 a	7.18 ± 1.24 a	5.51 ± 1.05 b
Psychrotrophic	7.13 ± 1.07 a	6.66 ± 1.18 a	7.02 ± 1.16 a	5.59 ± 1.14 b
Enterobacteria	4.23 ± 0.88 a	3.78 ± 1.35 a	3.96 ± 0.95 a	2.33 ± 0.51 b

Values (mean ± STD; *n* = 20) in the same row that share a letter show no significant differences between them (*p* > 0.05).

**Table 4:** Antibiotic resistance patterns of *L. monocytogenes* in untreated samples (controls). Data from Rodríguez-Melcón et al. [30].

Antibiotic Resistance Pattern	Number of Isolates in Control (Non-Treated) Samples		
	Spain	Portugal	Total
OX-FOX-FEP	0	1	1
OX-FOX-CTX-FEP	1	7	8
OX-FOX-CTX-FEP-SXT	0	1	1
OX-FOX-CTX-FEP-CIP	7	6	13
OX-FOX-CTX-FEP-F	3	3	6
OX-FOX-CTX-FEP-E	1	0	1
OX-FOX-CTX-FEP-RD	1	1	2
OX-FOX-CTX-FEP-SXT-CIP	2	0	2
OX-FOX-CTX-FEP-RD-CIP	3	0	3
OX-FOX-CTX-FEP-CIP-F	10	4	14
OX-FOX-CTX-FEP-E-RD	1	0	1
OX-FOX-CTX-FEP-SXT-CIP-F	2	0	2
OX-FOX-CTX-FEP-RD-CIP-F	5	1	6
OX-FOX-CTX-FEP-E-CIP-F	1	0	1
OX-FOX-CTX-FEP-E-RD-CIP	1	0	1
OX-FOX-CTX-FEP-SXT-TE-CIP-F	0	1	1
OX-FOX-CTX-FEP-CN-E-SXT-CIP	1	0	1
OX-FOX-CTX-FEP-E-RD-CIP-F	1	0	1
OX-FOX-CTX-FEP-SXT-CIP-ENR-F	1	0	1
OX-FOX-CTX-FEP-SXT-RD-CIP-F	3	0	3
OX-FOX-CTX-FEP-E-SXT-RD-TE	1	0	1

AMP (ampicillin, 10 µg), OX (oxacillin, 1 µg), FOX (cefoxitin, 30 µg), CTX (cefotaxime, 30 µg), FEP (cefepime, 30 µg), CN (gentamycin, 10 µg), E (erythromycin, 15 µg), VA (vancomycin, 30 µg), SXT (trimethoprim-sulfamethoxazole, 25 µg), RD (rifampicin, 5 µg), TE (tetracycline, 30 µg), C (chloramphenicol, 30 µg), CIP (ciprofloxacin, 5 µg), ENR (enrofloxacin, 5 µg), and F (nitrofurantoin, 300 µg).

**Table 5:** Antibiotic resistance patterns of *L. monocytogenes* in samples treated with sodium nitrite (SN).

Antibiotic Resistance Pattern	Number of Isolates in Samples Treated with SN		
	Spain	Portugal	Total
OX-FOX-CTX-FEP	0	2	2
OX-FOX-CTX-FEP-CIP	0	1	1
OX-FOX-CTX-FEP-F	3	2	5
OX-FOX-CTX-FEP-SXT	2	4	6
OX-FOX-CTX-FEP-RD	0	2	2
OX-FOX-CTX-FEP-CIP-F	12	6	18
OX-FOX-CTX-FEP-TE-F	2	0	2
OX-FOX-CTX-FEP-RD-CIP	1	0	1
AMP-OX-FOX-CTX-FEP-E	0	1	1
OX-FOX-CTX-FEP-RD-CIP-F	6	1	7
OX-FOX-CTX-FEP-E-CIP-F	2	0	2
OX-FOX-CTX-FEP-CN-SXT-F	1	0	1
OX-FOX-CTX-FEP-CN-RD-TE	1	0	1
OX-FOX-CTX-FEP-SXT-CIP-F	0	1	1
OX-FOX-CTX-FEP-CN-E-RD	0	1	1
OX-FOX-CTX-FEP-E-SXT-CIP-F	1	0	1
OX-FOX-CTX-FEP-SXT-RD-CIP-F	1	0	1
OX-FOX-CTX-FEP-RD-CIP-ENR-F	0	1	1
OX-FOX-CTX-FEP-CN-E-RD-TE	0	1	1
OX-FOX-CTX-FEP-CN-SXT-RD-TE-CIP	1	0	1
OX-FOX-CTX-FEP-CN-E-SXT-RD-TE	1	0	1
OX-FOX-CTX-FEP-CN-E-SXT-RD-CIP	0	1	1
OX-FOX-CTX-FEP-CN-E-SXT-RD-TE-CIP	1	0	1
OX-FOX-CTX-FEP-CN-E-SXT-RD-CIP-ENR	0	1	1

For interpretation, see Table 4.

**Table 6:** Antibiotic resistance patterns of *L. monocytogenes* in samples treated with nisin (Ni).

Antibiotic Resistance Pattern	Number of Isolates in Samples Treated with Ni		
	Spain	Portugal	Total
OX-FOX-CTX-FEP	0	3	3
OX-FOX-CTX-FEP-CIP	3	2	5
OX-FOX-CTX-FEP-RD	0	3	3
OX-FOX-CTX-FEP-F	3	4	7
OX-FOX-CTX-FEP-SXT	0	1	1
OX-FOX-CTX-FEP-CIP-F	15	3	18
OX-FOX-CTX-FEP-SXT-RD	0	1	1
OX-FOX-CTX-FEP-E-CIP	0	1	1
OX-FOX-CTX-FEP-RD-CIP-F	8	0	8
OX-FOX-CTX-FEP-CIP-ENR-F	1	0	1
OX-FOX-CTX-FEP-E-CIP-F	1	1	2
OX-FOX-CTX-FEP-SXT-RD-CIP-F	2	0	2
OX-FOX-CTX-FEP-E-SXT-CIP-F	1	0	1
OX-FOX-CTX-FEP-RD-CIP-ENR-F	1	0	1
OX-FOX-CTX-FEP-E-RD-CIP-F	1	0	1
OX-FOX-CTX-FEP-RD-C-CIP-F	1	0	1
AMP-OX-FOX-CTX-FEP-SXT-TE-F	0	1	1
OX-FOX-CTX-FEP-E-RD-TE-CIP	0	1	1
OX-FOX-CTX-FEP-CN-E-SXT-RD	0	1	1
OX-FOX-CTX-FEP-SXT-RD-CIP-ENR-F	1	0	1
OX-FOX-CTX-FEP-E-RD-TE-CIP-F	0	3	3
OX-FOX-CTX-FEP-CN-E-RD-TE-CIP-F	1	0	1
OX-FOX-CTX-FEP-CN-E-SXT-RD-CIP-ENR	0	1	1

For interpretation, see Table 4.

**Table 7:** Antibiotic resistance patterns of *L. monocytogenes* in samples treated with lactic acid (LA).

Antibiotic Resistance Pattern	Number of Isolates in Sampls Treated with LA		
	Spain	Portugal	Total
OX-FOX-CTX-FEP	0	6	6
OX-FOX-CTX-FEP-CIP	4	2	6
OX-FOX-CTX-FEP-F	2	0	2
OX-FOX-CTX-FEP-SXT	0	1	1
OX-FOX-CTX-FEP-RD	0	2	2
OX-FOX-CTX-FEP-CIP-F	5	3	8
OX-FOX-CTX-FEP-ENR-F	1	0	1
OX-FOX-CTX-FEP-RD-CIP	0	1	1
OX-FOX-CTX-FEP-SXT-CIP	0	1	1
OX-FOX-CTX-FEP-SXT-RD	0	1	1
OX-FOX-CTX-FEP-RD-F	0	1	1
OX-FOX-CTX-FEP-RD-CIP-F	3	2	5
AMP-OX-FOX-CTX-FEP-CIP-F	0	1	1
OX-FOX-CTX-FEP-SXT-CIP-F	0	1	1
OX-FOX-CTX-FEP-E-CIP-F	0	1	1
OX-FOX-CTX-FEP-CN-E-RD-TE	1	0	1
OX-FOX-CTX-FEP-SXT-RD-CIP-F	1	1	2
OX-FOX-CTX-FEP-E-RD-CIP-F	1	0	1
OX-FOX-CTX-FEP-E-TE-CIP-F	1	0	1
OX-FOX-CTX-FEP-CN-E-RD-TE-CIP	3	0	3
OX-FOX-CTX-FEP-CN-E-SXT-RD-CIP-F	1	0	1
OX-FOX-CTX-FEP-CN-E-RD-TE-ENR-F	1	0	1
OX-FOX-CTX-FEP-CN-E-SXT-RD-CIP-ENR	0	1	1
OX-FOX-CTX-FEP-CN-E-SXT-RD-TE-CIP-ENR-F	1	0	1

For interpretation, see Table 4.

## Data Availability

The data presented in this study are available on request from the corresponding author. The data are not publicly available due to confidentiality.
